# p*I*-Control in Comparative Fluorescence Gel Electrophoresis (CoFGE) using amphoteric azo dyes

**DOI:** 10.1016/j.dib.2015.03.007

**Published:** 2015-04-01

**Authors:** Marina Hanneken, Karel Šlais, Simone König

**Affiliations:** a Core Unit Proteomics, Interdisciplinary Center for Clinical Research, University of Münster, Germany; b Institute of Analytical Chemistry of the ASCR, v. v. i., Brno, Czech Republic

**Keywords:** 2D-PAGE, CoFGE, pI, Gel electrophoresis

## Abstract

Amphoteric azo dyes were used for internal control of p*I* values in Comparative two-dimensional Fluorescence Gel Electrophoresis (CoFGE) [1]. The 2D-gel images of separated *Escherichia coli* proteins as well as those of colored amphoteric dyes separated by isoelectric focussing are presented. The latter were used to correct for variation in the first electrophoretic dimension and further improve protein coordinate assignment in 2D-gel electrophoresis. Data tables are supplied to demonstrate p*I*-value calibration and the effect on the assignment of protein spot coordinates.

Specifications table

**Table:** 

Subject area	*Biochemistry*

More specific subject area	*Protein analysis, proteomics, protein gel electrophoresis*
Type of data	*Table, 2D-gel images, figure*
How data was acquired	*Comparative 2D Fluorescence Gel Electrophoresis (CoFGE) with FlatTop Tower (Serva Electrophoresis GmbH)*
Data format	*Typhoon 9400 images, raw and analyzed*
Experimental factors	*Replicate experiments using E. coli, internal molecular weight standard and pI-control*[1-4]
Experimental features	*Proof-of-principle experiments for improvement of CoFGE (pI-control)*
Data source location	*Münster, Germany*
Data accessibility	*Data is with this article*

Value of the data

• Comparative Fluorescence Gel Electrophoresis CoFGE allows reproducible protein spot assignment based on a reference grid formed by an internal molecular weight standard (*y*-dimension).
• Amphoteric azo dyes control p*I* (*x*-dimension) completing the CoFGE toolkit.

## 1 Experimental design, materials and methods

Amphoteric azo dyes were used for the control of the first dimension (p*I*) in horizontal Comparative two-dimensional Fluorescence Gel Electrophoresis (hCoFGE) [1]. CoFGE itself uses an internal reference grid formed by internal protein standards to correct for the gel-to-gel variation in the second dimension of 2D polyacrylamide GE improving protein spot coordinate assignment [2–4].

## 2 Data

### 2.1 p*I*-Control

**Fig. 1:**
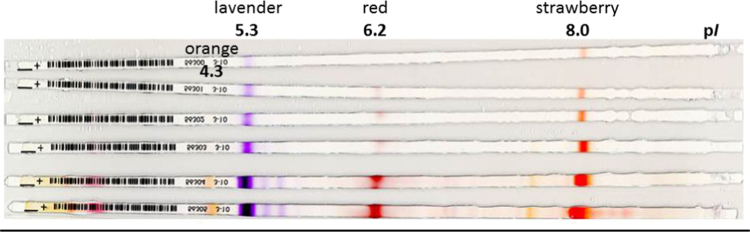
Low-molecular weight p*I*-markers for CoFGE [1]. Amounts per color tested from bottom to top: 1; 0.5; 0.25; 0.2; 0.1; 0.025 µg. Immobiline TM Dry Strip pH 3–10, 24 cm, GE Healthcare.

Amphoteric azo dyes were synthesized and used as low-molecular weight p*I*-markers for CoFGE [1]. The application range was 0.025 to 1 µg per Immobiline TM Dry Strip (pH 3–10, 24 cm, GE Healthcare, Fig. 1).

### 2.2 Replicate CoFGE experiments for method validation

**Fig. 2:**
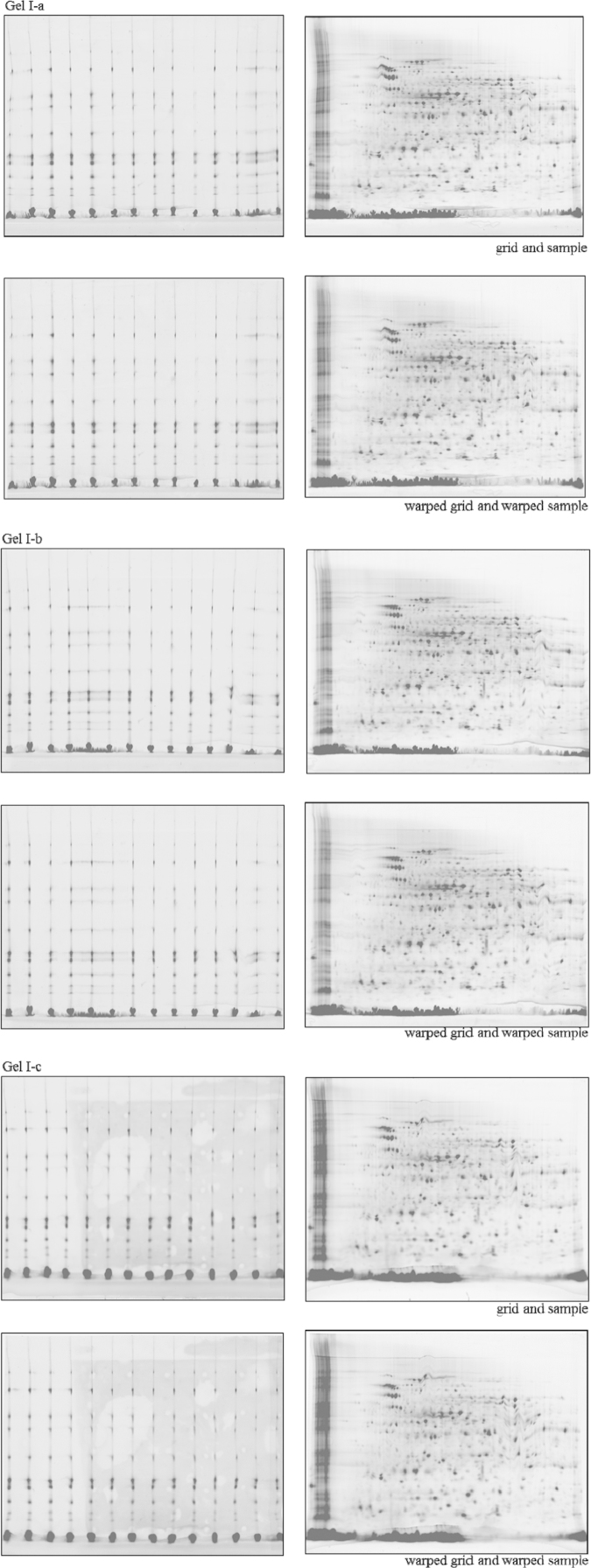
Three comparative fluorescence gel electrophoresis experiments (Gel I-a, I-b and I-c). Shown are images of reference protein grid mixture versus *E. coli* sample run on one gel without p*I*-control. Each gel was scanned immediately using Typhoon9400 at 560 pmt. *E. coli* lysate was labeled with G-Dye300 and the reference proteins with G-Dye200 (NH DyeAgnostics, Halle, Germany). The protein mix was loaded into the 14 self-made O-wells about 2 mm above the p*I* strip (24 cm).

**Fig. 3:**
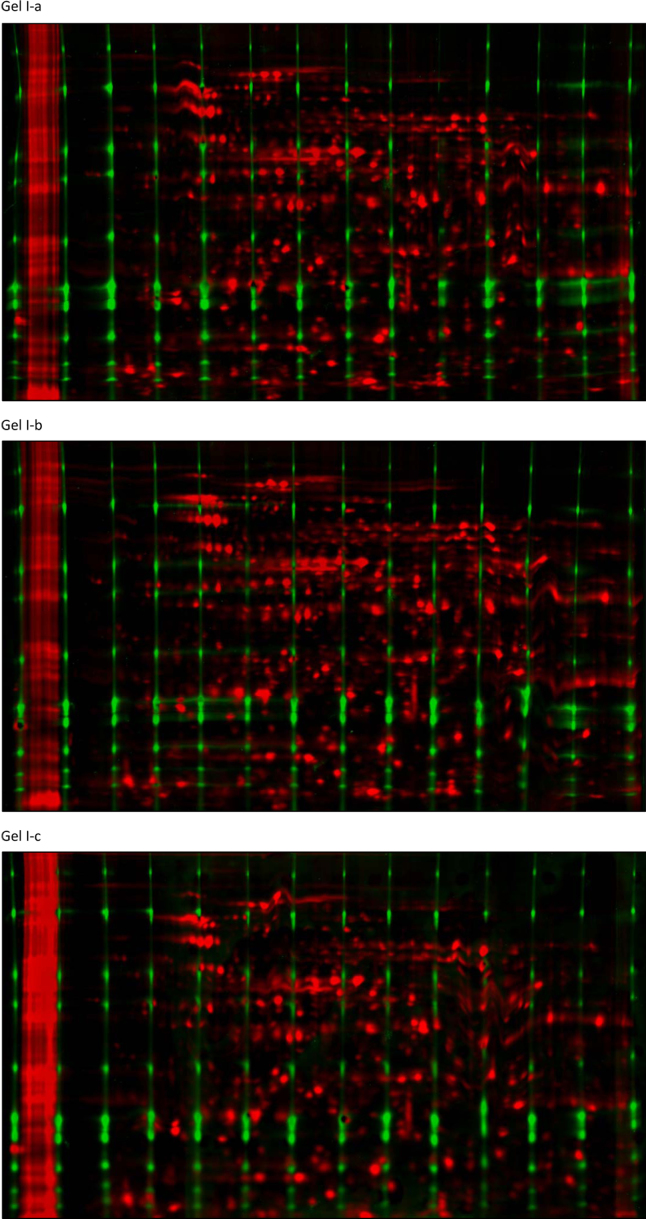
False color overlays from Delta 2D (Gel I-a, Gel I-b, Gel I-c, first experiment, without p*I*-control). Unwarped grid (green) vs. sample (MW-warped, red).

**Fig. 4:**
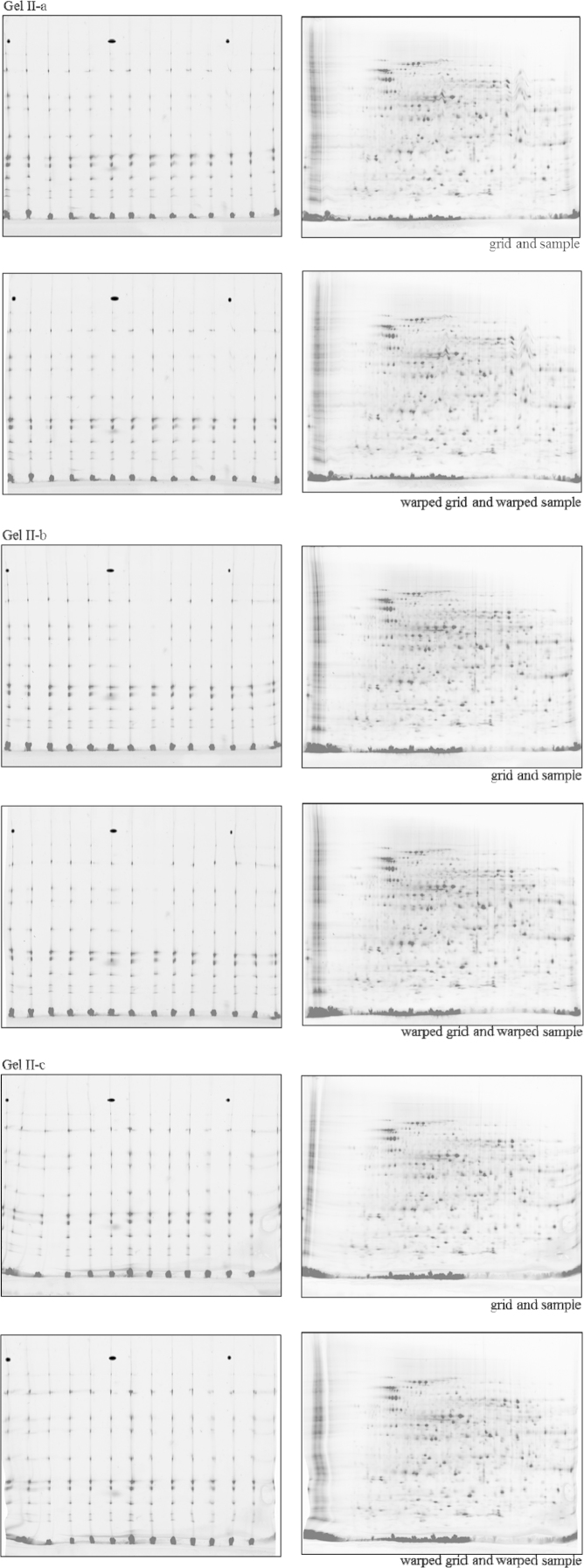
Three comparative fluorescence gel electrophoresis experiments (Gel II-a, II-b and II-c). Shown are images of reference protein grid mixture versus *E. coli* sample run on one gel with p*I*-control. Each gel was scanned immediately using Typhoon9400 at 560 pmt. *E. coli* lysate was labeled with G-Dye300 and the reference proteins with G-Dye200. The protein mix was loaded into the 14 self-made O-wells about 2 mm above the p*I* strip (24 cm).

**Fig. 5:**
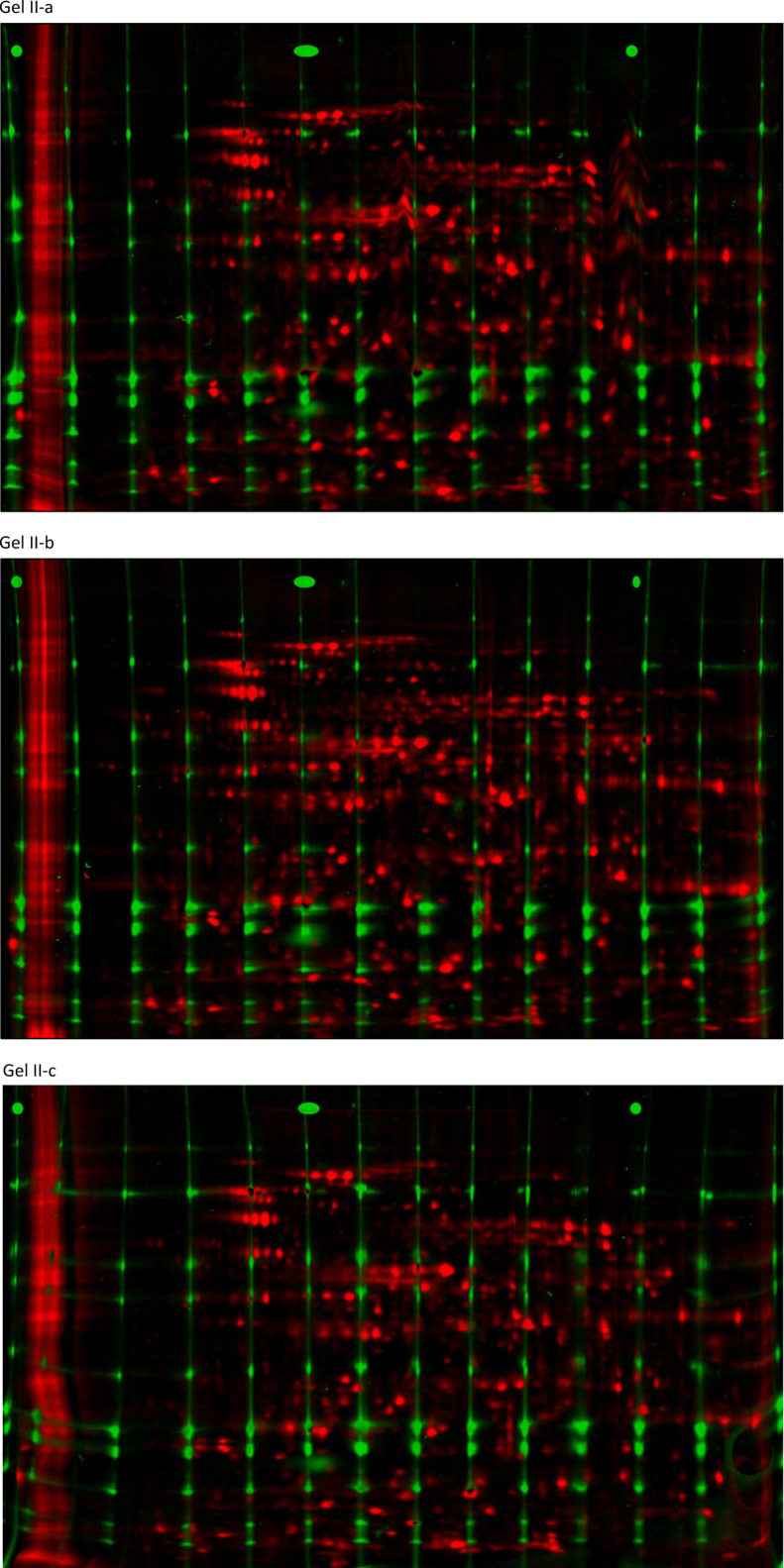
False color overlays from Delta 2D (Gel II-a, Gel II-b, Gel II-c, second experiment, with p*I*-control). Unwarped grid (green) vs sample (MW- and p*I*-warped, red).

Three CoFGE experiments each were performed without (Fig. 2; Gel I-a, I-b and 1-c) and with p*I*-control (Fig. 4; Gel II-a, II-b and II-c). Shown are images of reference protein grid mixture versus *E. coli* sample run on one gel before and after warping with Delta 2D (Decodon). Each gel was scanned using Typhoon9400 at 560 pmt. *E. coli* lysate was labeled with G-Dye300 and the reference proteins with G-Dye200 (NH DyeAgnostics, Halle, Germany). The protein mix was loaded into 14 self-made O-wells about 2 mm above the p*I* strip. Figs. 3 and 5 present the corresponding false color overlays for illustration. Tables 1 and 2 deliver the respective mean and deviation from mean in percent for coordinates of selected protein spots in the comparative experiments with and without MW-warping against the marker protein grid.

## Figures and Tables

**Table 1:** Mean and deviation from mean in percent for coordinates of selected protein spots in three comparative experiments (first experiment, without p*I*-control, Gel I-a, I-b and I-c) with and without MW-warping against the marker protein grid.

**Mean and deviation from mean for spot coordinates**				
	**No warping**		**Warping**	
**Spot-No.**	***x***	***y***	***x***	***y***
**1**	1.48	3.13	1.56	1.51
**2**	0.79	3.86	1.20	0.52
**3**	1.16	3.94	1.32	1.01
**4**	0.93	3.81	0.98	0.93
**5**	2.01	2.60	2.32	0.47
**6**	1.47	2.14	1.06	0.41
**7**	0.42	1.43	0.94	1.13
**8**	3.26	1.54	2.62	0.11
**9**	2.42	1.52	2.00	0.28
**10**	0.49	1.26	0.81	0.69
**11**	0.66	3.88	0.58	0.63
**12**	2.37	1.32	1.69	0.44
**13**	2.34	0.88	1.89	0.21
**14**	1.20	1.34	1.06	0.70
**15**	1.18	1.04	1.43	1.17
**16**	1.22	3.44	1.16	0.38
**17**	3.67	0.11	1.80	0.63
**18**	1.73	0.76	1.10	0.66
**19**	3.10	0.16	2.42	0.15
**20**	1.99	0.18	1.48	0.15
**21**	1.40	1.00	1.16	0.61
**22**	0.60	1.48	0.90	0.33
**23**	0.32	1.87	0.40	0.48
**24**	3.84	2.14	1.54	0.73
**25**	4.23	0.33	2.08	0.37
**26**	5.83	0.44	2.11	0.08
**27**	3.94	0.78	1.02	0.29
**28**	5.15	1.06	1.98	0.73
**29**	3.02	2.37	2.04	0.51
**30**	0.99	2.05	1.04	0.18
**Mean (%)**	**2.11**	**1.73**	**1.46**	**0.55**
**Range (%)**	**0.32–5.83**	**0.11–3.94**	**0.4–2.62**	**0.08–1.51**

**Table 2:** Mean and deviation from mean in percent for coordinates of selected protein spots in three comparative experiments (second experiment, with p*I*-control, Gel II-a, II-b and II-c) with and without warping against the marker protein grid and additionally p*I*-warping against azo p*I*-markers.

**Mean and deviation from mean for spot coordinates**				
	**No warping**		**Warping**	
**Spot-no.**	***x***	***y***	***x***	***y***
**1**	2.12	0.55	2.14	0.84
**2**	1.80	0.44	2.37	0.86
**3**	1.05	0.24	1.93	0.46
**4**	0.99	1.34	1.88	0.84
**5**	0.38	0.14	1.01	0.47
**6**	1.50	0.48	0.56	0.64
**7**	1.24	0.70	1.64	0.74
**8**	1.31	0.34	1.14	0.55
**9**	1.71	0.76	1.95	0.51
**10**	0.84	0.81	1.34	0.79
**11**	1.38	0.77	1.19	0.88
**12**	1.97	0.61	0.42	0.78
**13**	1.56	0.52	0.75	0.55
**14**	1.16	0.56	2.27	0.08
**15**	0.99	0.61	2.19	0.46
**16**	1.38	1.05	0.77	0.34
**17**	1.26	0.51	1.60	0.21
**18**	1.72	0.72	1.03	0.44
**19**	1.70	0.48	0.49	0.18
**20**	1.21	0.52	1.86	0.12
**21**	1.18	0.77	2.13	0.16
**22**	1.26	0.92	0.55	0.10
**23**	0.12	1.65	0.07	0.73
**24**	1.75	0.51	0.72	0.50
**25**	1.08	0.47	0.72	0.20
**26**	0.30	0.23	0.20	0.11
**27**	0.72	0.18	0.74	0.28
**28**	4.56	0.30	2.39	0.09
**29**	1.55	0.80	0.86	0.76
**30**	1.19	2.26	0.41	0.30
**Mean (%)**	**1.37**	**0.67**	**1.24**	**0.47**
**Range (%)**	**0.12–4.56**	**0.14–2.26**	**0.07–2.39**	**0.08–0.88**

## References

[1] M. Hanneken, K. Šlais, S. König p*I*-Control in Comparative Fluorescence Gel Electrophoresis (CoFGE) using Amphoteric Azo Dyes EuPA Open Proteomics, 2015, http://dx.doi.org/10.1016/j.euprot.2015.03.003. 10.1016/j.dib.2015.03.007 PMC4510154 26217748

[2] Ackermann D., Wang W., Streipert B., Geib B., Grün L., König S. (2012). Comparative fluorescence two-dimensional gel electrophoresis using a gel strip sandwich assembly for the simultaneous on-gel generation of a reference protein spot grid Electrophoresis.

[3] D. Ackermann, W. Wang, L. Grün, S. König Improved gel electrophoresis Patent application EP11167383.6, May 25, 2011; WO 2012/159769 A1 Nov. 29, 2012; Patent No. 12729346.2-1554 May 25, 2012, publication number EP 2 715 331.

[4] Hanneken M., König S. (2014). Horizontal comparative fluorescence two-dimensional gel electrophoresis (hCoFGE) for improved spot coordinate detection Electrophoresis.

